# Computational Modeling of Glucose Transport in Pancreatic β-Cells Identifies Metabolic Thresholds and Therapeutic Targets in Diabetes

**DOI:** 10.1371/journal.pone.0053130

**Published:** 2012-12-27

**Authors:** Camilla Luni, Jamey D. Marth, Francis J. Doyle

**Affiliations:** 1 Department of Chemical Engineering, University of California Santa Barbara, Santa Barbara, California, United States of America; 2 Sansum Diabetes Research Institute, Santa Barbara, California, United States of America; 3 Center for Nanomedicine, Sanford-Burnham Medical Research Institute and the Department of Molecular, Cellular and Developmental Biology, University of California Santa Barbara, Santa Barbara, California, United States of America; National Research Council of Italy (CNR), Italy

## Abstract

Pancreatic β-cell dysfunction is a diagnostic criterion of Type 2 diabetes and includes defects in glucose transport and insulin secretion. In healthy individuals, β-cells maintain plasma glucose concentrations within a narrow range in concert with insulin action among multiple tissues. Postprandial elevations in blood glucose facilitate glucose uptake into β-cells by diffusion through glucose transporters residing at the plasma membrane. Glucose transport is essential for glycolysis and glucose-stimulated insulin secretion. In human Type 2 diabetes and in the mouse model of obesity-associated diabetes, a marked deficiency of β-cell glucose transporters and glucose uptake occurs with the loss of glucose-stimulated insulin secretion. Recent studies have shown that the preservation of glucose transport in β-cells maintains normal insulin secretion and blocks the development of obesity-associated diabetes. To further elucidate the underlying mechanisms, we have constructed a computational model of human β-cell glucose transport in health and in Type 2 diabetes, and present a systems analysis based on experimental results from human and animal studies. Our findings identify a metabolic threshold or “tipping point” whereby diminished glucose transport across the plasma membrane of β-cells limits intracellular glucose-6-phosphate production by glucokinase. This metabolic threshold is crossed in Type 2 diabetes and results in β-cell dysfunction including the loss of glucose stimulated insulin secretion. Our model further discriminates among molecular control points in this pathway wherein maximal therapeutic intervention is achieved.

## Introduction

Glucose transport across the plasma membrane is an essential process among cells and organisms [1], [2], [3]. Glucose is a major source of metabolic energy, yet deviations of glucose concentration from a narrow range in the blood of mammals can be life threatening. A chronic elevation of blood glucose concentration is linked to the pathology of diabetes. Normally, pancreatic β-cells sense a postprandial rise in blood glucose and secrete insulin into circulation by a process termed glucose-stimulated insulin secretion (GSIS). The resulting activation of insulin receptors among peripheral tissues increases glucose uptake in normalizing blood glucose levels [4]. In this way, the pancreatic β-cell acts in concert with peripheral insulin action to regulate glucose homeostasis in the organism. The ability of the β-cell to transport glucose across the plasma membrane and thereby sense changes in blood glucose concentration is an essential component of normal β-cell function and the maintenance of glucose homeostasis.

In Type 2 diabetes (T2D), β-cells appear defective in sensing glucose, and this has recently been linked with diminished expression of both GLUT-1 and GLUT-2 glucose transporters [5], [6]. Deficiency of glucose transporter expression and glucose uptake among normal β-cells causes β-cell dysfunction with loss of the GSIS response [7]. A similar study in mice administered a high-fat diet indicated that diminished β-cell Glut-2 expression contributed to disease pathogenesis, while preservation of β-cell glucose transport and GSIS prevented β-cell failure and the onset of obesity-associated diabetes [8]. Those studies further reported a conserved sequence of molecular events in human and mouse β-cells initiated by elevated levels of free fatty acids, transmitted by nuclear exclusion and down-modulation of HNF1A and FOXA2 transcription factors, and affected by GNT-4A glycosyltransferase deficiency. These events were found to diminish expression of GLUT-1 and GLUT-2 with markedly reduced glucose transport and loss of GSIS, and revealed that an acquired deficiency of β-cell glucose transport promotes the pathogenesis of diabetes.

Glucokinase (GK) activity is normally the limiting factor in β-cell glucose utilization [9]. Upon entering the β-cell, glucose is rapidly phosphorylated by GK forming glucose-6-phosphate (G6P). This ensures that glucose cannot exit the β-cell through the same diffusive glucose transporters GLUT-1 and GLUT-2, and instead can enter glycolysis. Intracellular concentrations of G6P normally increase in response to elevated blood glucose. This promotes glycolysis and subsequent events including the GSIS response. The inheritance of partial defects in GK activity by gene mutation impedes the formation of G6P and disables the GSIS response, as observed in the human disease known as Mature Onset Diabetes of the Young, MODY2 [10].

In understanding how the acquisition of deficient β-cell glucose transport may contribute to the pathogenesis of Type 2 diabetes, we have developed a mathematical model of glucose transport that integrates experimental findings that include human data from β-cells of normal and T2D donors [7], with supporting data from rodent studies. This model includes the GLUT-1 and GLUT-2 glucose transporters of human β-cells as well as components of a molecular pathway that controls their expression [7]. Our findings indicate a physiological and metabolic threshold exists below which glucose entry, and not GK activity, is rate limiting in G6P production. Among β-cells isolated from animal models of diabetes and human T2D donors, we show that β-cell glucose transport is below this threshold while healthy humans and rodents maintain glucose transport well above the threshold. We further identify molecular nodes within this pathogenic pathway where therapeutic intervention would be most effective.

## Results

### Initial Steps in GSIS

Glucose transport into the β-cell occurs by facilitated diffusion through plasma membrane-resident GLUT-1 and GLUT-2. While Glut-2 is the main transporter in mouse β-cells and is essential for GSIS, in human β-cells both GLUT-1 and GLUT-2 are present and it appears that either can support GSIS [7], [11], [12], [13], [14], [15]. Both transporters exhibit Michaelis-Menten kinetics with different 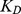 values for glucose indicating the concentration of glucose when the rate is half of the maximum velocity. This is approximately 3 mM for GLUT-1 and 17 mM for GLUT-2 [16]. Despite these differences, expression of either transporter alone can promote sufficient glucose entry for phosphorylation by GK and the GSIS response [7], [15], [17]. The GK rate is described by Hill kinetics with a 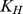 of 8 mM [18] and an exponent 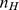 of 1.7 [9]. The parameter 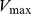 in the Michaelis-Menten and Hill kinetics is dependent on the level of expression of the glucose transporters and GK. We therefore expressed 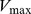 of GLUT-1 and GLUT-2 as:
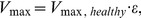
(1)where 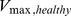 is the parameter value for normal β-cells, and 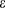 represents the fraction of plasma membrane-resident glucose transporter expression compared to normal. Thus, 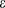 is equal to unity in normal cells, and less than unity in β-cells from T2D donors in which the glucose transporters are diminished. We assumed identical GK activities in β-cells from T2D donors and normal human β-cells. The complete system of equations for this element of the model is reported in (Text S1).

**Figure 1:**
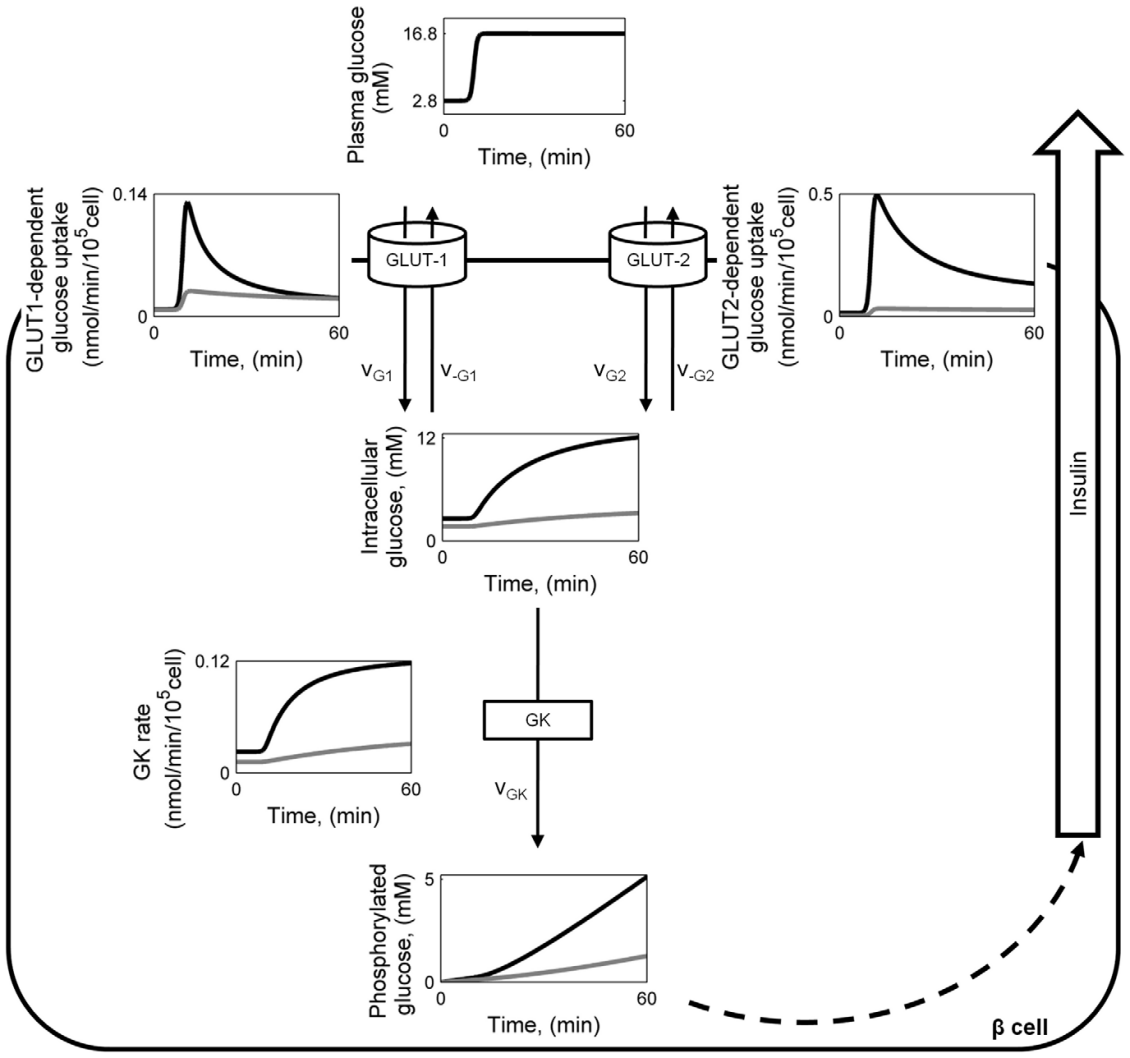
Intracellular kinetics of the first steps of pancreatic β-cell glycolysis in health and T2D. A change in plasma glucose concentration from 2.8 mM to 16.8 mM was applied as an input to the model. Net GLUT1-dependent glucose uptake was calculated as the difference between inwards and outwards rates, 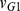 and 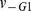. Net GLUT2-dependent uptake was calculated analogously. Phosphorylated glucose concentration includes also the concentration of its derivates in the pathway that are not explicitly modeled. Black lines refer to the behavior of healthy β-cells, gray lines represent behavior of β-cells from T2D donors.

We simulated the first stages of glucose uptake and utilization in normal β-cells and from two T2D donors whose average plasma membrane-resident GLUT-1 and GLUT-2 are markedly reduced to 14% and 5% of normal, respectively [7]. This simulation included a postprandial glucose excursion from 2.8 mM to 16.8 mM (Figure 1), which corresponds to the concentration range used in previous experiments [7]. The net glucose uptake by the cell is given by the difference between the inward and the outward flow of unphosphorylated glucose through GLUT-1 and GLUT-2. The qualitative behavior of the two glucose transporters is similar: when plasma glucose is increased to 16.8 mM, glucose enters the β-cell at a high rate because the extra-cellular glucose concentration is greatly above equilibrium considering the intracellular concentration. The glucose uptake rate is progressively compensated by the export rate until a steady-state is reached at a higher glucose concentration. The result is a rise in net glucose transport through each transporter until this new steady-state is established. Due to the lower 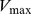 of GLUT-1, GLUT-2 accounts for most of the glucose uptake when both transporters are present. The kinetics (i.e., time-dependent behavior) of glucose transport through the two transporters is also different. In addition, because of the lower 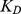 value of GLUT-1, the relative difference between the steady-states at high and low blood glucose is smaller for this transporter (Figure 1).

The diminished β-cell surface expression of GLUT-1 and GLUT-2 in T2D can strongly affect glycolysis, even in the presence of normal GK activity. Since intracellular glucose concentration is much lower than normal, the GK rate and G6P accumulation are also strongly reduced (Figure 1).

### Metabolic Threshold

**Figure 2:**
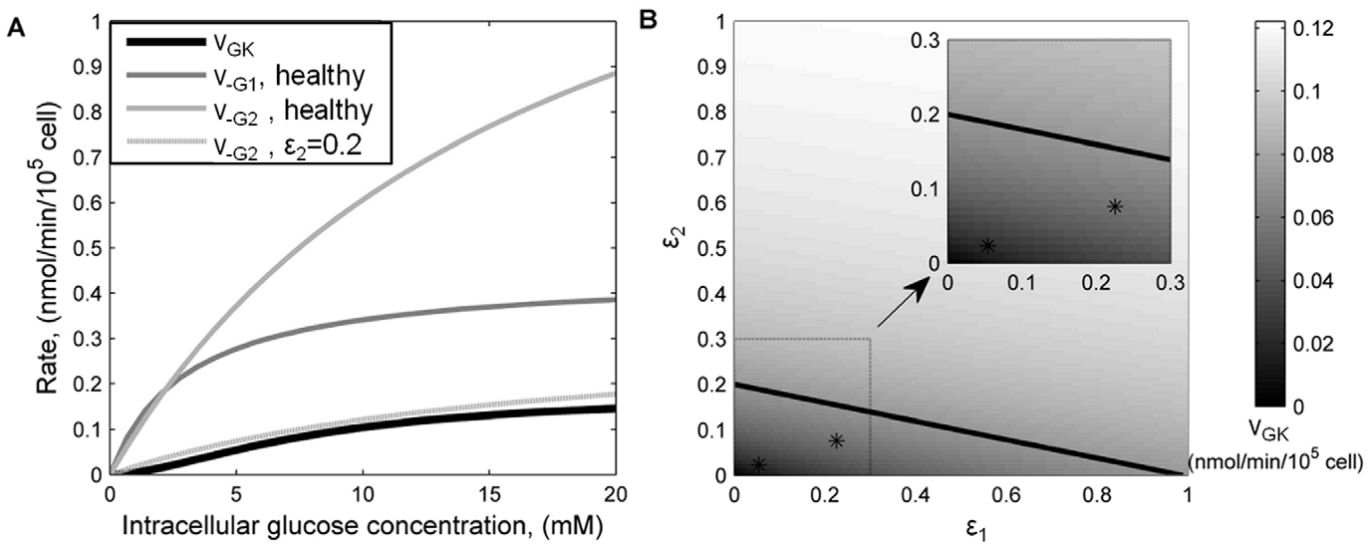
Comparison of glucose transport and phosphorylation in health and T2D β-cells. (A) GLUT-1 and GLUT-2 outwards rates (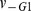 and 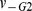, respectively), and GK kinetics as a function of intra-cellular glucose concentration for normal (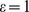) glucose transporters’ expression and reduced GLUT-2 expression (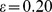). (B) Steady-state GK rate calculated at 16.8 mM extra-cellular glucose concentration as a function of concerted GLUT-1 and GLUT-2 deficiency. ε_1_ and ε_2_ are the fractions of GLUT-1 and GLUT-2, respectively, compared to normal. The solid line indicates the threshold between transport- and phosphorylation-limited G6P formation (derived in (A)), whereas asterisks indicate the positions of T2D patients β-cells. Inset represents an enlargement of the lower left region.

Once inside the β-cell, intracellular glucose is subject to two competitive mechanisms: outward diffusion through glucose transporters and phosphorylation by GK. The GK rate has been calculated to be a slow enzymatic step that normally is rate-limiting for insulin secretion [9]. For a range of intracellular glucose concentrations, we compared the GK rate of G6P production with the outward diffusion rates of glucose through GLUT-1 and GLUT-2 in a healthy β-cell (Figure 2A). The results confirm that the glucose phosphorylation rate by GK is much slower than the outward glucose transport through both GLUT-1 and GLUT-2 under normal conditions. This means that after glucose enters the cell most of it diffuses out because GK phosphorylation is relatively slow. Thus GK is the glucose sensor and rate-limiting factor in G6P formation among healthy β-cells. However, when β-cell surface expression of GLUT-2 is approximately 20% of normal (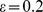 in equation (1)), its outwards transport rate is comparable to the GK rate (Figure 2A). Thus, in a β-cell expressing 20% of normal GLUT-2, and in the absence of GLUT-1, there is a transition in the controlling mechanism forming G6P. Below this threshold of GLUT-2 expression, glucose transport becomes the rate-limiting factor.

We evaluated the steady-state G6P production rate at reduced levels of glucose transporter expression when extra-cellular glucose concentration is elevated to 16.8 mM, in order to determine when the GK rate falls below the calculated threshold. We used the model to simulate the concerted reductions of GLUT-1 and GLUT-2 at the β-cell plasma membrane. The results are shown in Figure 2B, where a healthy β-cell is represented at the top right corner, with a GK rate of 0.12 nmol/min/10^5^ cells. The T2D patients previously studied [7] are represented in the lower left region. In β-cells expressing 20% GLUT-2 and no GLUT-1, the threshold condition identified in Figure 2A, intracellular glucose concentration is about 6.5 mM and GK rate is 0.07 nmol/min/10^5^ cells. This intracellular glucose concentration is 50% of normal and consequently the GK rate is approximately 60% of normal. This further specifies the critical threshold or tipping point when transition occurs from GK-controlled to glucose transport-controlled G6P formation. In Figure 2B, we highlighted all the possible combinations of GLUT-1 and GLUT-2 expression that produce the same critical GK rate. Strikingly, data points from the T2D patients are located below this threshold. These findings further agree with experimental data [7] and indicate that glucose transport by GLUT-1 can compensate for the absence of membrane GLUT-2 leaving GK as a glycolytic pacemaker when GLUT-1 is expressed at normal levels (Figure 2B).

### Regulation of GLUT-1 and GLUT-2 Expression

**Figure 3:**
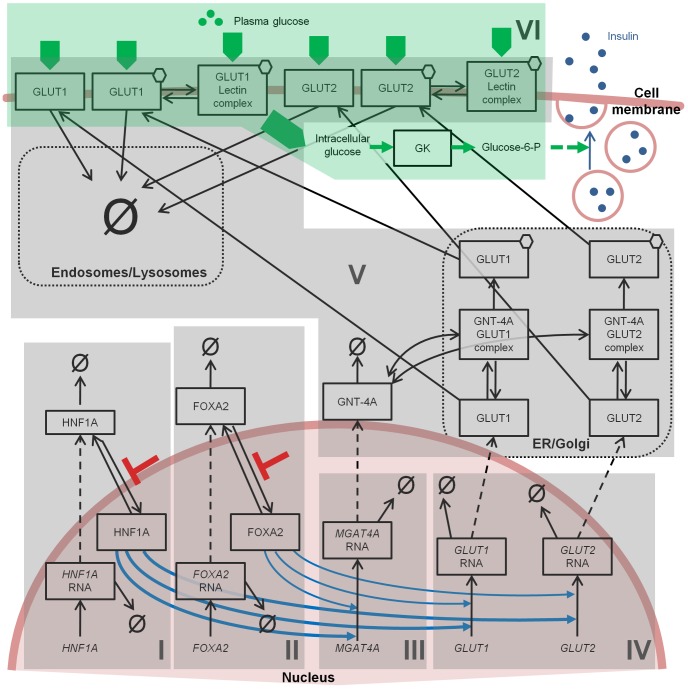
Schematic representation of the processes included in the mathematical model. The six subsystems discussed in the text are highlighted and denoted by roman numbers. Thick blue arrows indicate activation of transcription by promoter binding and histone hyperacetylation, thin blue arrows activation only by promoter binding; red bars indicate an inhibitory effect on nuclear residency of transcription factors. Ø symbol indicates degradation, hexagons the glycosylated forms of the proteins. Green arrows show the path of glucose entrance into the cell, its phosphorylation, and the ultimate activation of insulin secretion.

The model above describes glucose entry into the human pancreatic β-cell and its accumulation following phosphorylation by GK. Considering that diabetes can be induced in animal models by β-cell glucose transporter deficiency, we integrated the previous human model (modules VI in Figure 3) with factors involved in the transcriptional and post-translational regulation of GLUT-1 and GLUT-2 (Figure 3). Experimental data obtained from human β-cell studies of normal donors, T2D donors, and palmitic acid-treated normal β-cells, supported model development [7] (Methods). Results from rodent studies were also integrated where indicated. As experimental data were obtained from a pool of cells, the single-cell model we developed is representative of average β-cell behavior, neglecting the intrinsic heterogeneity of the cell population. Stochastic noise is also averaged in the experimental cell population, further justifying our deterministic modeling approach.

The full model includes the regulation of *GLUT1* and *GLUT2* genes by the transcription factors HNF1A and FOXA2. Specifically, we described the production and degradation of HNF1A and FOXA2 at the RNA and protein level, and the translocation of the two proteins to the nucleus where they transactivate their target genes (modules I and II in Figure 3) [8], [19]. Besides *GLUT1* and *GLUT2*, HNF1A and FOXA2 also regulate *MGAT4A*, another gene particularly relevant for β-cell glucose entry, as discussed below. The transcription of these three genes, *GLUT1*, *GLUT2* and *MGAT4A*, includes two layers of regulation: first, HNF1A induces histone hyperacetylation at target gene promoter nucleosomes [7], [8], [19], [20]; and second, HNF1A and FOXA2 bind to target gene promoter sequences and promote transcription [7], [8], [21] (modules III and IV).

GLUT-1 and GLUT-2 are regulated also at the post-translational level, by protein glycosylation. In particular, glucose transporter residency at the β-cell plasma membrane requires a specific N-glycan structure produced on both transporters by the Golgi-resident GNT-4A glycosyltransferase enzyme, the product of *MGAT4A* gene, [7], [8], [22] (module V). This post-translational modification promotes GLUT-1 and GLUT-2 interaction with one or more lectins at the plasma membrane and maintains their residency at the membrane by a mechanism competing with normal endocytic internalization and degradation rates. Thus, despite cycles of production and degradation, GLUT-1 and GLUT-2 glycoproteins are steadily present at the β-cell plasma membrane in healthy individuals [23]. The model includes these post-translational regulation steps, as schematically shown in Figure 3, where GLUT-1 and GLUT-2 are simply identified as ‘glycosylated’ or ‘unglycosylated’, according to the presence or absence of the GNT-4A-dependent N-glycan modification, although N-glycosylation comprises multiple different N-glycan structures on the glucose transporters. We assumed the same kinetic rates for GLUT-1 and GLUT-2 interactions with MGAT4A and lectins. Thus, in the model, differences in the concentration of the two transporters at the membrane are the result of differences in transcription.

Within the network considered, our previous experimental work showed that both β-cells from T2D donors and β-cells from healthy donors treated with palmitic acid exhibit reduced glucose transporter expression, diminished glucose uptake and impaired G6P production, compared to normal β-cells [7]. Underlying this functional impairment there are multiple network disruptions including decreased HNF1A and FOXA2 nuclear localization, reduced transcription of the *MGAT4A*, *GLUT1* and *GLUT2* genes, and decreased abundance of plasma membrane-resident glucose transporters [7]. We verified that the model was able to capture all these experimental observations by simply perturbing the network at the level of HNF1A and FOXA2 translocation to the nucleus (Figure 3, red arrows). Introducing an inhibitory factor acting on these translocations produces, by itself, all the other observed alterations as well as the impairment of glucose transport.

**Figure 4:**
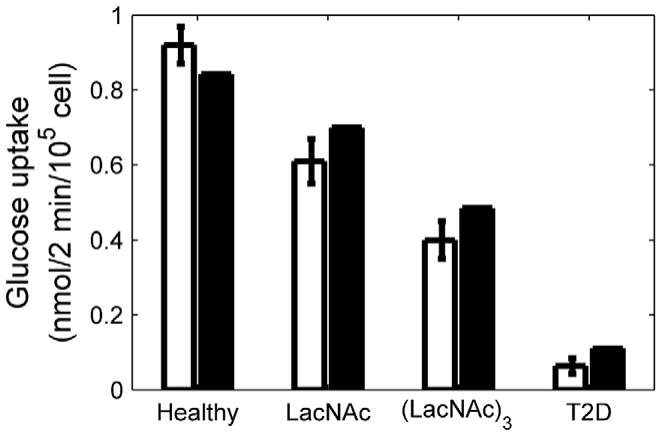
Glucose uptake as a function of membrane glucose transporters’ expression. Comparison between experimental data (white) (Ohtsubo et al., 2011) and model results (black) of glucose uptake at 10 mM extracellular glucose concentration, for β-cells from healthy and T2D patients, and for cells from healthy donors treated with LacNAc and (LacNAc)_3_, as indicated. Error bars represent standard deviation of the data. The percentage of GLUT-1 and GLUT-2 expression compared to healthy β-cells is also indicated in each case.

The complete model structure, parameter values and comparisons between model results and human experimental data are provided in the (Text S2), and are summarized in the Methods section. We also verified the model with glucose uptake measurements from normal β-cells co-cultured with LacNAc and (LacNAc)_3_ glycans [7] (Figure 4). These glycans compete with the GNT-4A-glycosylated glucose transporters for binding to β-cell lectins that promote cell surface residency, thereby resulting in reduced expression of GLUT-1 (75% of normal with LacNAc and 57% with (LacNAc)_3_) and GLUT-2 (80% and 48%, respectively).

In healthy human β-cells, GLUT-1 is the predominantly expressed transporter; however, GLUT-2 is also expressed at lower level [7], [12], [24]. From these and other observations, we assumed that, in healthy human β-cells, ∼80% of plasma membrane glucose transporters are GLUT-1 and ∼20% are GLUT-2. Nevertheless, the higher value of 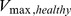 for GLUT-2 means that a single molecule of GLUT-2 transports more glucose than a single molecule of GLUT-1. Therefore, GLUT-2 accounts for the majority of glucose transport even if it is expressed at much lower levels than GLUT-1. Simulating glucose transporter expression in a β-cell from a T2D donor, by inhibiting HNF1A and FOXA2 translocation to the nucleus, without further modifications, we calculated that in disease conditions 92% of the glucose transporters present at the β-cell surface are GLUT-1 and 8% are GLUT-2. Thus, also in human T2D β-cells, GLUT-1 remains the most abundant transporter at plasma membrane.

### Control Point Identification

The full mathematical model provides a link between glucose transporter expression and specific intracellular biological components affecting their residency at the cell membrane. Thus, points of this regulatory network that are more sensitive targets of therapeutic intervention can be investigated to identify best strategies for restoring normal glucose transport and β-cell function.

**Figure 5:**
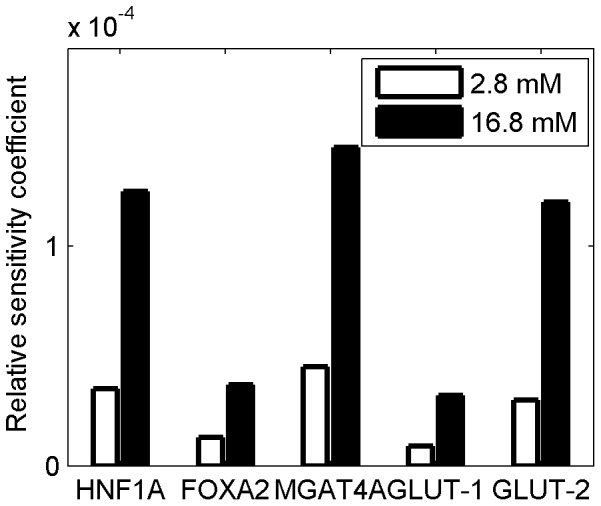
Therapeutic sensitivity analysis among pathway components. Steady-state sensitivity of GK rate in T2D cells with respect to elevation in the RNA abundance of the genes indicated, at two plasma glucose concentrations, as shown in the legend. The sensitivity coefficients are normalized with respect to the GK rate and abundance of the RNAs among β-cells.

We used the T2D β-cell model of reduced glucose transport due to the effect of nuclear exclusion of HNF1A and FOXA2 (red arrows in Figure 3). We measured the effect on the GK rate when perturbing different points in the network by a local sensitivity analysis. A high sensitivity to perturbation suggests a more effective point for potential therapeutic intervention because larger increases in glucose transport leading to GSIS are produced. Perturbations were applied to the RNA content of *HNF1A*, *FOXA2*, *MGAT4A*, *GLUT1* and *GLUT2*, singularly. Propagation of these changes to the GK rate of G6P formation was calculated at 2.8 and 16.8 mM blood glucose concentrations. The results are shown in terms of normalized sensitivity coefficients, as defined in Methods section, to produce a fair comparison between the effects of different perturbations (Figure 5).

The model indicates highest sensitivity to the perturbation of *MGAT4A* RNA, in comparison with perturbation of RNAs encoding the other proteins. This finding remains valid at different extracellular glucose concentrations, but is more critical at higher glucose concentrations when the effect of these perturbations is emphasized. The sensitivity of the GK rate to HNF1A and FOXA2 is mediated by the role they play as transcriptional activators of *MGAT4A*, *GLUT1*, and *GLUT2*. Because HNF1A also affects histone acetylation, the impact of an intervention on *HNF1A* RNA expression is much more pronounced than an intervention involving *FOXA2* RNA expression. A change in *GLUT2* RNA has a higher impact on GK rate than the same percentage change in *GLUT1* RNA because, even if much less abundant, GLUT-2 has a higher 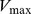 and accounts for most of glucose uptake.

## Discussion

Deficient pancreatic β-cell glucose transport has been found to be a key pathogenic feature of a pathway to diabetes that incorporates multiple factors linked in a sequence of molecular events [7]. We have developed a computational model for β-cell glucose transport and the alterations that occur in health and diabetes from the published findings of multiple laboratories. Our simulations closely approximated the experimental data and provide insights including the presence of a metabolic threshold or “tipping point” in β-cell glucose uptake and utilization. While GK is normally rate limiting in G6P production and β-cell glucose utilization, our model indicates that glucose transport across the plasma membrane becomes rate limiting among models of obesity-associated diabetes in rodents and human β-cells from donors with T2D. The consequence of disrupting β-cell glucose transport across the plasma membrane appears most severe during increased concentrations of glucose typical of postprandial blood glucose excursions that normally lead to the GSIS response. In modeling multiple approaches to increase glucose transporter expression to prevent disease onset, we have detected different sensitivities to intervention. The most effective therapeutic perturbation indicated by the model and supported by experimental data is not achieved by a direct increase in glucose transporter expression *per se*, but instead by conservation of the GNT-4A glycosyltransferase enzyme that supports β-cell glucose transporter residency at the plasma membrane.

The switch in control of G6P formation from GK activity to glucose transport in diabetes results from diminished expression of both GLUT-1 and GLUT-2 glucose transporters at the β-cell plasma membrane. Human β-cells express both GLUT-1 and GLUT-2, however, GLUT-2 appears to be responsible for the majority of glucose transport. Diminished glucose transporter expression reaches a critical threshold at various combinations of GLUT-1 and GLUT-2 deficiencies. When less than 20% of normal GLUT-2 expression occurs in absence of GLUT-1, for example, glucose transport becomes rate limiting in the formation of G6P, which correlates to approximately 60% of the normal GK rate. Although the precise “tipping point” varies with the relative levels of GLUT-1 and GLUT-2, our modeling indicates that this threshold is greatly exceeded in β-cells isolated from animal models of obesity-associated diabetes and human T2D donors. Median decrements of β-cell surface glucose transporter expression in T2D reached more than 85% for each transporter. Substantial decrements of GLUT-2 expression appear required to abolish GSIS, while normal expression of GLUT-1 can compensate for the absence of GLUT-2 and restore GSIS. This is consistent with the deficiency of both GLUTs along with GSIS in T2D β-cells and recent findings that unaltered expression of either GLUT-1 or GLUT-2 is sufficient to maintain GSIS in normal β-cells [7].

The computational model that we constructed revealed markedly different sensitivities to molecular perturbations among different pathway components. Preservation of the β1-4GlcNAc glycan linkage produced by the GNT-4A glycosyltransferase is predicted to be most effective at maintaining glucose transporter expression at the β-cell surface and maintaining normal glucose transport with GSIS activity. This feature of the model is consistent with experimental data showing that increased *Glut2* gene expression in mouse β-cells cannot similarly maintain normal glucose transporter expression and inhibits all disease signs in obese mice [6]. The reason appears to reflect the diminished half-lives at the β-cell surface of misglycosylated GLUT-1 and GLUT-2 in the absence of sufficient GNT-4A activity. This misglycosylation results in the absence of the glycan ligand of cell surface resident lectins, including one or more of the galectins [8]. Even with low levels of *GLUT* RNA expression, sufficient *MGAT4A*-encoded GNT-4A activity and normal GLUT glycosylation can maintain β-cell surface GLUT expression sufficiently to support GSIS. The sensitivities of the model to FOXA2 and HNF1A are also different. Higher sensitivity of HNF1A is predicted due to its additional role in modulating chromatin histone acetylation. The increase of FFA and, specifically, palmitic acid was also included in the model, but was limited to its inhibitory effect on HNF1A and FOXA2 translocation to the nucleus. Thus, the model would capture a beneficial effect of FFA reduction on glucose uptake. However, down-modulation of FFA receptor binding and signaling was not incorporated into our model. It has been reported that FFA receptors are essential to normal β-cell function [25]. An efficacious inhibitor of FFA receptor signaling may disrupt the normal functions of multiple cell types. In contrast, chronic elevations of FFAs may become pathogenic [26], perhaps by membrane fusion in receptor independent processes among cell organelles including mitochondria, leading to increased oxidative stress with changes to intracellular transcriptional networks.

From computational modeling of the initial steps in pancreatic β-cell glycolysis, beginning with glucose transport and G6P formation, we have achieved an integration of experimental and computational data that encompasses a recently discovered pathogenic pathway to T2D that proceeds through attenuation of pancreatic β-cell GNT-4A activity and glucose transport [7]. This model closely simulates outcomes of rigorous experimentation in rodents and human β-cells, and further indicates the presence of a metabolic threshold wherein the pacemaker of β-cell glycolysis switches from GK activity to glucose transport. Beyond this tipping point, β-cells are defective in glucose sensing and are unable to produce G6P by mechanisms that support normal functions including GSIS. Our model is further consistent with studies of human β-cells from T2D donors, in which this metabolic threshold is crossed and GSIS is defective. With inherent metabolic flexibility, β-cells can subsequently respond by increasing their oxidation of fatty acids as a substitute source of cellular energy. However, this flexibility cannot restore normal insulin secretion processes linked to glucose sensing, thereby inducing a pathogenic pathway leading to diabetes.

## Methods

### Model Description

The mathematical model was developed as a system of ordinary differential equations. Most of the biochemical kinetic rates were expressed by mass action laws. Exceptions are the GLUT-1 and GLUT-2 transport described by Michaelis-Menten kinetics, and the GK rate and glucose transporters interaction with lectins described by Hill kinetics. Further details and MATLAB programs are provided in (Text S1, S2 and S3).

### Sensitivity Analysis

Relative sensitivity coefficients, 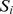, were calculated approximating by finite differences the expression 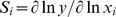, where 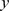 represents GK rate at steady-state for a given extra-cellular glucose concentration, and 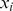 the concentration of RNA perturbed, for 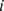 corresponding to *HNF1A*, *FOXA2*, *MGAT4A*, *GLUT1*, and *GLUT2*.

## Supporting Information

Text S1 **Detailed description of the reduced mathematical model.**
(PDF)

Text S2 **Detailed description of the full model.**
(PDF)

Text S3 **MATLAB programs of the full model.**
(PDF)
