# Supplementary material for: Computational Modeling of Glucose Transport in Pancreatic β-Cells Identifies Metabolic Thresholds and Therapeutic Targets in Diabetes
Source: PLoS One. 2012 Dec 27;7(12):e53130. doi: 10.1371/journal.pone.0053130 (PMC3531366; doi:10.1371/journal.pone.0053130)
Supplement: Text S1 — Detailed description of the reduced mathematical model. (PDF) [file pone.0053130.s001.pdf]

Supplemental Information, Text S1

Luni C, Marth JD, Doyle III FJ. Computational Modeling of Glucose Transport in Pancreatic β-cells Identifies Metabolic Thresholds and Therapeutic Targets in Diabetes. *PLOS One*.

First steps in GSIS. The system of equations for glucose transport and phosphorylation in a β-cell is given by the following expressions, with nomenclature and parameters as indicated below:

$$d[Gluc]_{i.c.}/dt = v_{G1} + v_{G2} - v_{-G1} - v_{-G2} - v_{GK},$$

where:

$$v_{G1} = \frac{V_{\max,1}[Gluc]_{e.c.}}{K_{D,1} + [Gluc]_{e.c.}}$$

$$v_{G2} = \frac{V_{\max,2}[Gluc]_{e.c.}}{K_{D,2} + [Gluc]_{e.c.}}$$

$$v_{-G1} = \frac{V_{\max,1}[Gluc]_{i.c.}}{K_{D,1} + [Gluc]_{i.c.}}$$

$$v_{-G2} = \frac{V_{\max,2}[Gluc]_{i.c.}}{K_{D,2} + [Gluc]_{i.c.}}$$

$$v_{GK} = \frac{V_{\max,GK}[Gluc]_{i.c.}^{n_H}}{K_H^{n_H} + [Gluc]_{i.c.}^{n_H}}$$

$$V_{\max,1} = V_{\max,1healthy} \cdot \varepsilon_1$$

$$V_{\max,2} = V_{\max,2healthy} \cdot \varepsilon_2$$

$$\varepsilon_1 = \frac{[mGlut1]}{[mGlut1]_{healthy}}$$

$$\varepsilon_2 = \frac{[mGlut2]}{[mGlut2]_{healthy}}$$

GLUT-1 and GLUT-2 are present at the cell membrane in three forms (as specified in the model described in Text S2): basic, glycosylated, and glycosylated within a lectin-bound complex. We assumed that glycosylation does not affect glucose transport kinetics, but only the residence time at the membrane of the glucose transporters.

<u>Nomenclature.</u>

v_{G1} = rate of extra-cellular glucose entrance into the β-cell through GLUT-1, (mM/min)

v_{G2} = rate of extra-cellular glucose entrance into the β-cell through GLUT-2, (mM/min)

v_{-G1} = rate of intra-cellular glucose exit out of the β-cell through GLUT-1, (mM/min)

v_{-G2} = rate of intra-cellular glucose exit out of the β-cell through GLUT-2, (mM/min)

v_{GK} = rate of intra-cellular glucose phosphorylation by GK, (mM/min)

$[Gluc]_{i.c.}$ = intracellular glucose concentration, (mM)

$[Gluc]_{e.c.}$ = extra-cellular glucose concentration, (mM)

$[mGlut1]$ = steady-state membrane GLUT-1 concentration

$[mGlut2]$ = steady-state membrane GLUT-2 concentration

$_{healthy}$ = for β-cell in normal healthy conditions

ε_1 = fraction of $[mGlut1]$ respect to normal

ε_2 = fraction of $[mGlut2]$ respect to normal

<u>Parameters.</u>

Parameter	*Value*	*Unit*	*Reference*
$V_{\max,1 healthy}$	$1059.54 \cdot 10^{-3}$	mM/min*	**
$K_{D,1}$	3	mM	Uldry *et al.* (2002)
$V_{\max,2 healthy}$	$3910.51 \cdot 10^{-3}$	mM/min*	**
$K_{D,2}$	17	mM	Uldry *et al.* (2002)
$V_{\max,GK}$	$420.17 \cdot 10^{-3}$	mM/min*	**
K_H	8	mM	Davis *et al.* (1999)
n_H	1.7		Matschinsky (1996)
ε_1, T2D β-cell	0.14		Ohtsubo *et al.* (2011)
ε_2, T2D β-cell	0.05		Ohtsubo *et al.* (2011)

* Assuming the volume of intracellular space is equal to $4.2\ pl/cell$.

** Least-squares fitting of data in Figure 3i in Ohtsubo *et al.* (2011).
