# Supplementary material for: Computational Modeling of Glucose Transport in Pancreatic β-Cells Identifies Metabolic Thresholds and Therapeutic Targets in Diabetes
Source: PLoS One. 2012 Dec 27;7(12):e53130. doi: 10.1371/journal.pone.0053130 (PMC3531366; doi:10.1371/journal.pone.0053130)
Supplement: Text S2 — Detailed description of the full model. (PDF) [file pone.0053130.s002.pdf]

Supplemental Information, Text S2

Luni C, Marth JD, Doyle III FJ. Computational Modeling of Glucose Transport in Pancreatic β-cells Identifies Metabolic Thresholds and Therapeutic Targets in Diabetes. *PLOS One*.

Model Equations. The complete mathematical model for modules I-V is given by the following system of equations:

$$
\begin{cases}
\dot{x}_1 = v_1 - v_{26} \\
\dot{x}_2 = v_2 - v_3 - v_{16,1} - v_{16,2} + v_{17,1} + v_{17,2} \\
\dot{x}_3 = v_4 - v_{27} \\
\dot{x}_4 = v_5 - v_6 + v_7 - v_8 \\
\dot{x}_5 = v_6 - v_7 \\
\dot{x}_6 = v_9 - v_{28} \\
\dot{x}_7 = v_{10} - v_{11} + v_{12} - v_{13} \\
\dot{x}_8 = v_{11} - v_{12} \\
\dot{x}_{9,1} = v_{14,1} - v_{29,1} \\
\dot{x}_{10,1} = v_{15,1} - v_{16,1} + v_{17,1} - v_{19,1} \\
\dot{x}_{11,1} = v_{18,1} - v_{20,1} \\
\dot{x}_{12,1} = v_{19,1} - v_{22,1} \\
\dot{x}_{13,1} = v_{20,1} - v_{21,1} - v_{23,1} + v_{30,1} \\
\dot{x}_{16,1} = v_{21,1} - v_{30,1} \\
\dot{x}_{17,1} = v_{16,1} - v_{17,1} - v_{18,1} \\
\dot{x}_{9,2} = v_{14,2} - v_{29,2} \\
\dot{x}_{10,2} = v_{15,2} - v_{16,2} + v_{17,2} - v_{19,2} \\
\dot{x}_{11,2} = v_{18,2} - v_{20,2} \\
\dot{x}_{12,2} = v_{19,2} - v_{22,2} \\
\dot{x}_{13,2} = v_{20,2} - v_{21,2} - v_{23,2} + v_{30,2} \\
\dot{x}_{16,2} = v_{21,2} - v_{30,2} \\
\dot{x}_{17,2} = v_{16,2} - v_{17,2} - v_{18,2}
\end{cases}
$$

The model variables, x_i, are defined in Table S1 and the kinetic expressions are given in Table S2, where k_i's represent model kinetic parameters, and α_i's are constant factors that describe the inhibitory effect of palmitic acid and T2D. Symbols x_i and v_i are also represented in Figure S1.

φ_i's are expressions that include the contribution of transcription factors on RNA synthesis, and are defined as follows (1):

$$\varphi_i = \frac{w_{i_0} + w_{i_5}\frac{x_5}{K_{i_5}} + w_{i_8}\frac{x_8}{K_{i_8}} + w_{i_{58}} r_{i_{58}} \frac{x_5}{K_{i_5}}\frac{x_8}{K_{i_8}}}{1 + \frac{x_5}{K_{i_5}} + \frac{x_8}{K_{i_8}} + r_{i_{58}}\frac{x_5}{K_{i_5}}\frac{x_8}{K_{i_8}}}$$

where K_{i_j} represent the equilibrium transcription factor-promoter dissociation constants, r_{i_j} the ratio of K_{i_j}'s when transcription factor j (either HNF1A or FOXA2) is bound to a promoter alone and in the presence of the other transcription factor, $0 \le w_i \le 1$ are modulation coefficients subject to the following relationship:

$$w_{i_{58}} = 1 - w_{i_0} - w_{i_5} - w_{i_8}\ .$$

Modules I-V of the model were solved at steady-state with Mathematica (Wolfram Research Inc.), the analytical expressions for the state variables were then implemented in MATLAB (The MathWorks) and integrated with equations of Module VI. The complete MATLAB model is reported as Text S3.

Model Parameters. The human experimental data are summarized in Table S3 (2). As they are given by ratios between different component concentrations at steady-state, an absolute value for the model parameters could not be identified. Thus, the model was scaled, according to a dimensional analysis (3), with an arbitrary concentration of RNA, x_{RNA}, and protein, x_{pr}, and a time factor, τ. The ratio x_{pr}/x_{RNA} was also fitted to the experimental data. However, all the results presented are independent from this scaling.
The fraction of *MGAT4A* with bound HNF1A, y_{1_5}, FOXA2, y_{1_8}, and both, $y_{1_{58}}$, are defined by the following expressions (1):

$$y_{1_5} = \frac{\frac{x_5}{K_{1_5}}}{1 + \frac{x_5}{K_{1_5}} + \frac{x_8}{K_{1_8}} + r_{1_{58}}\frac{x_5}{K_{1_5}}\frac{x_8}{K_{1_8}}}$$

$$y_{1_8} = \frac{\frac{x_8}{K_{1_8}}}{1 + \frac{x_5}{K_{1_5}} + \frac{x_8}{K_{1_8}} + r_{1_{58}}\frac{x_5}{K_{1_5}}\frac{x_8}{K_{1_8}}}$$

$$y_{1_{58}} = \frac{r_{1_{58}} \dfrac{x_5}{K_{1_5}} \dfrac{x_8}{K_{1_8}}}{1 + \dfrac{x_5}{K_{1_5}} + \dfrac{x_8}{K_{1_8}} + r_{1_{58}} \dfrac{x_5}{K_{1_5}} \dfrac{x_8}{K_{1_8}}}$$

An analogous definition holds for transcription factors bound to GLUT-1 and GLUT-2 genes. Parameter estimation was performed by a two-step approach: first, a heuristic search method, the genetic algorithm, was used to span the parameter space randomly, avoiding local minima; then, a simplex search method, implemented by the *fmincon* function in MATLAB, was applied in order to minimize the cost function locally. The cost function was defined as the sum of least-square deviations of model outputs from experimental data, weighted by the experimental variability of each data. The outcome of the parameter estimation procedure included multiple sets of parameter values that were scored according to the value of the cost function. The parameter set having the lowest cost function value, reported in Table S4, was used in all computations in the Main Text. The comparison of model output using this parameter set and the experimental data is shown in Figure S2.

Sensitivity Analysis. The sensitivity analysis results, shown in Figure 5 in the Main Text, were investigated for robustness to uncertainty in parameter values (Figure S3). A normally distributed noise, with standard deviation of 10% (according to the mean width of the confidence intervals of the parameters), was added to the parameter values to obtain 200 parameter sets, half perturbing a single parameter (Figure S3 A) and half perturbing 10 random parameters at once (Figure S3 B). The relative sensitivity coefficients were calculated with the different parameter sets to investigate the effect of a change in the RNA content of *HNF1A*, *FOXA2*, *MGAT4A*, *GLUT1* and *GLUT2* genes on the steady-state GK rate, at an extra-cellular glucose concentration of 16.8 mM.

Figure S3 shows the results of the sensitivity analysis. The trend of Figure 5 is confirmed in both cases (Figure S3 A and B), with a higher standard deviation in Figure S3 B respect to Figure S3 A. In each case the relative sensitivity coefficients were compared using one-way ANOVA with Tukey post-test, with $p<0.05$ indicating significance. Each pair comparison resulted significant, showing that the sensitivity analysis shown in Figure 5 is robust to both single and multiple parameter uncertainty.

The sensitivity analysis above was performed using small changes in the parameters to calculate the sensitivity coefficients by numerically approximating the partial derivatives around the steady-state. Since pharmacological interventions would introduce much larger changes in the network, we also performed a sensitivity analysis for larger changes in parameter values, 25% of their nominal value, to capture the nonlinear behavior of the network. Figure S4 shows the results of this analysis and confirms the trend seen in Figure S3 B, indicating highest sensitivity to the perturbation of *MGAT4A* RNA. However, this analysis only partially supports the

identification of the most promising pharmacological targets, because such large changes may induce secondary network responses not accounted for in the presented model structure.

References

1. Schilstra MJ, et al. (2002) The logic of gene regulation. In *Proceedings of 3rd International Conference on Systems Biology (ICSB): The Logic of Life, Karolinska Institutet, Stockholm, Sweden*.
2. Ohtsubo K, et al. (2011) Pathway to diabetes through attenuation of pancreatic beta cell glycosylation and glucose transport. *Nat Med* 17:1067-U1162.
3. Barenblatt GI. (1996) Scaling, self-similarity, and intermediate asymptotics. Cambridge University Press, Cambridge, UK.

Table S1. Nomenclature of model variables.

x_1	MGAT4A RNA
x_2	GNT4A protein
x_3	HNF1A RNA
x_4	HNF1A extra-nuclear protein
x_5	HNF1A nuclear protein
x_6	FOXA2 RNA
x_7	FOXA2 extra-nuclear protein
x_8	FOXA2 nuclear protein
$x_{9,1}$	GLUT-1 RNA
$x_{10,1}$	GLUT-1 protein, ER/Golgi, non-glycosylated
$x_{11,1}$	GLUT-1 protein, ER/Golgi, glycosylated
$x_{12,1}$	GLUT-1 protein, membrane, non-glycosylated
$x_{13,1}$	GLUT-1 protein, membrane, glycosylated
$x_{16,1}$	GLUT-1 protein, membrane, glycosylated, lectin-bound
$x_{17,1}$	GNT4A-GLUT-1 complex
$x_{9,2}$	GLUT-2 RNA
$x_{10,2}$	GLUT-2 protein, ER/Golgi, non-glycosylated
$x_{11,2}$	GLUT-2 protein, ER/Golgi, glycosylated
$x_{12,2}$	GLUT-2 protein, membrane, non-glycosylated
$x_{13,2}$	GLUT-2 protein, membrane, glycosylated
$x_{16,2}$	GLUT-2 protein, membrane, glycosylated, lectin-bound
$x_{17,2}$	GNT4A-GLUT-2 complex

Table S2. Kinetic expressions.

$v_1 = k_1^{'}\varphi_1 \frac{x_5}{k_1^{"} + x_5}$ (*)	$v_{17,2} = k_{17}x_{17,2}$
$v_2 = k_2 x_1$	$v_{18,1} = k_{18}x_{17,1}$
$v_3 = k_3 x_2$	$v_{18,2} = k_{18}x_{17,2}$
$v_4 = k_4$	$v_{19,1} = k_{19}x_{10,1}$
$v_5 = k_5 x_3$	$v_{19,2} = k_{19}x_{10,2}$
$v_6 = k_6\alpha_6 x_4$	$v_{20,1} = k_{20}x_{11,1}$
$v_7 = k_7 x_5$	$v_{20,2} = k_{20}x_{11,2}$
$v_8 = k_8 x_4$	$v_{21,1} = \frac{k_{21}^{'} x_{13,1}^{n_{21}}}{(k_{21}^{"})^{n_{21}} + x_{13,1}^{n_{21}}}$ (*)
$v_9 = k_9$	$v_{21,2} = \frac{k_{21}^{'} x_{13,2}^{n_{21}}}{(k_{21}^{"})^{n_{21}} + x_{13,2}^{n_{21}}}$ (*)
$v_{10} = k_{10}x_6$	$v_{22,1} = k_{22}x_{12,1}$
$v_{11} = k_{11}\alpha_{11}x_7$	$v_{22,2} = k_{22}x_{12,2}$
$v_{12} = k_{12}x_8$	$v_{23,1} = k_{23}x_{13,1}$
$v_{13} = k_{13}x_7$	$v_{23,2} = k_{23}x_{13,2}$
$v_{14,1} = k_{14,1}^{'}\varphi_{14,1} \frac{x_5}{k_{14,1}^{"} + x_5}$ (*)	$v_{26} = k_{26}x_1$
$v_{14,2} = k_{14,2}^{'}\varphi_{14,2} \frac{x_5}{k_{14,2}^{"} + x_5}$ (*)	$v_{27} = k_{27}x_3$
$v_{15,1} = k_{15,1}x_{9,1}$	$v_{28} = k_{28}x_6$
$v_{15,2} = k_{15,2}x_{9,2}$	$v_{29,1} = k_{29,1}x_{9,1}$
$v_{16,1} = k_{16}x_2 x_{10,1}$	$v_{29,2} = k_{29,2}x_{9,2}$

$v_{16,2} = k_{16} x_2 x_{10,2}$	$v_{30,1} = k_{30} x_{16,1}$
$v_{17,1} = k_{17} x_{17,1}$	$v_{30,2} = k_{30} x_{16,2}$

(*) Superscripts ' and " are used to distinguish parameters having the same subscript, which indicates the kinetic expression.

Table S3. Experimental data.

(Legend: ^{norm} healthy β-cells; ^{palm} palmitic acid-treated cells; $^{\mathrm{T2D}}$ β-cells from T2D patients)

Experiment No.	Description	Figures from (2)
I.1	$x_5^{norm}/(x_4^{norm}+x_5^{norm})$	Fig. 2c
I.2	$x_5^{palm}/(x_4^{palm}+x_5^{palm})$	Fig. 2c
I.3	$x_5^{\mathrm{T2D}}/(x_4^{\mathrm{T2D}}+x_5^{\mathrm{T2D}})$	Fig. 3a
II.1	$x_8^{norm}/(x_7^{norm}+x_8^{norm})$	Fig. 2c
II.2	$x_8^{palm}/(x_7^{palm}+x_8^{palm})$	Fig. 2c
II.3	$x_8^{\mathrm{T2D}}/(x_7^{\mathrm{T2D}}+x_8^{\mathrm{T2D}})$	Fig. 3a
III.1	x_1^{palm}/x_1^{norm}	Fig. 2f
III.2	$x_1^{\mathrm{T2D}}/x_1^{norm}$	Fig. 3b
III.3	$(y_{1_8}^{palm}+y_{1_{58}}^{palm})/(y_{1_8}^{norm}+y_{1_{58}}^{norm})$	Fig. 2d
III.4	$(y_{1_5}^{palm}+y_{1_{58}}^{palm})/(y_{1_5}^{norm}+y_{1_{58}}^{norm})$	Fig. 2e
IV.1	$x_{9,1}^{palm}/x_{9,1}^{norm}$	Fig. 2f
IV.2	$x_{9,1}^{\mathrm{T2D}}/x_{9,1}^{norm}$	Fig. 3b
IV.3	$(y_{14,1_8}^{palm}+y_{14,1_{58}}^{palm})/(y_{14,1_8}^{norm}+y_{14,1_{58}}^{norm})$	Fig. 2d
IV.4	$(y_{14,1_5}^{palm}+y_{14,1_{58}}^{palm})/(y_{14,1_5}^{norm}+y_{14,1_{58}}^{norm})$	Fig. 2e
IV.5	$x_{9,2}^{palm}/x_{9,2}^{norm}$	Fig. 2f
IV.6	$x_{9,2}^{\mathrm{T2D}}/x_{9,2}^{norm}$	Fig. 3b
IV.7	$(y_{14,2_8}^{palm}+y_{14,2_{58}}^{palm})/(y_{14,2_8}^{norm}+y_{14,2_{58}}^{norm})$	Fig. 2d
IV.8	$(y_{14,2_5}^{palm}+y_{14,2_{58}}^{palm})/(y_{14,2_5}^{norm}+y_{14,2_{58}}^{norm})$	Fig. 2e
V.1	$\left(x_{12,1}^{\mathrm{T2D}}+x_{13,1}^{\mathrm{T2D}}+x_{16,1}^{\mathrm{T2D}}\right)\Big/\left(x_{12,1}^{norm}+x_{13,1}^{norm}+x_{16,1}^{norm}\right)$	Fig. 3d
V.2	$\left(x_{12,2}^{\mathrm{T2D}}+x_{13,2}^{\mathrm{T2D}}+x_{16,2}^{\mathrm{T2D}}\right)\Big/\left(x_{12,2}^{norm}+x_{13,2}^{norm}+x_{16,2}^{norm}\right)$	Fig. 3d

Table S4. Parameter values.

$k_1^{'} = 6.17 \cdot 10^{-6}\ x_{RNA}\ \ \tau^{-1}$	$K_{14,1_8} = 1.91 \cdot 10^{-10}\ x_{pr}$
$w_{1_0} = 1.79 \cdot 10^{-11}$	$k_{14,1}^{''} = 4.02 \cdot 10^{-12}\ x_{pr}$
$w_{1_5} = 1.79 \cdot 10^{-20}$	$k_{14,2}^{'} = 2.27 \cdot 10^{-8}\ x_{RNA}\ \ \tau^{-1}$
$w_{1_8} = 0.033$	$w_{14,2_0} = 6.57 \cdot 10^{-11}$
$r_{1_{58}} = 2.55 \cdot 10^{-9}$	$w_{14,2_5} = 6.10 \cdot 10^{-14}$
$K_{1_5} = 1.40 \cdot 10^{-9}\ x_{pr}$	$w_{14,2_8} = 1.27 \cdot 10^{-11}$
$K_{1_8} = 3.04 \cdot 10^{-11}\ x_{pr}$	$r_{14,2_{58}} = 9.35 \cdot 10^{-19}$
$k_1^{''} = 1.11 \cdot 10^{-11}\ x_{pr}$	$K_{14,2_5} = 1.51 \cdot 10^{-8}\ x_{pr}$
$k_2 = 2.58 \cdot 10^{-15}\ \tau^{-1} x_{RNA}^{-1} x_{pr}$	$K_{14,2_8} = 3.88 \cdot 10^{-20}\ x_{pr}$
$k_3 = 1.23 \cdot 10^{-17}\ \tau^{-1}$	$k_{14,2}^{''} = 1.09 \cdot 10^{-5}\ x_{pr}$
$k_4 = 4.16 \cdot 10^{-12}\ x_{RNA}\ \ \tau^{-1}$	$k_{15,1} = 2.54 \cdot 10^{-13}\ \tau^{-1} x_{RNA}^{-1} x_{pr}$
$k_5 = 4.69 \cdot 10^{-12}\ \tau^{-1} x_{RNA}^{-1} x_{pr}$	$k_{15,2} = 3.11 \cdot 10^{-1}\ \tau^{-1} x_{RNA}^{-1} x_{pr}$
$k_6 = 0.016\ \tau^{-1}$	$k_{16} = 8.87 \cdot 10^{-16}\ x_{pr}^{-1}\ \ \tau^{-1}$
$\alpha_{6,palm} = 0.148$	$k_{17} = 5.72 \cdot 10^{-2}\ \tau^{-1}$
$\alpha_{6,\mathrm{T2D}} = 0.109$	$k_{18} = 6.36 \cdot 10^{-10}\ \tau^{-1}$
$k_7 = 5.14 \cdot 10^{-3}\ \tau^{-1}$	$k_{19} = 3.30 \cdot 10^{-10}\ \tau^{-1}$
$k_8 = 6.85 \cdot 10^{-8}\ \tau^{-1}$	$k_{20} = 2.46 \cdot 10^{-5}\ \tau^{-1}$
$k_9 = 3.45 \cdot 10^{-19}\ x_{RNA}\ \ \tau^{-1}$	$k_{21}^{'} = 6.98 \cdot 10^{-16}\ \tau^{-1} x_{pr}$
$k_{10} = 7.08 \cdot 10^{-16}\ \tau^{-1} x_{RNA}^{-1} x_{pr}$	$k_{21}^{''} = 1.6 \cdot 10^{-18}\ x_{pr}$
$k_{11} = 1.53 \cdot 10^{-14}\ \tau^{-1}$	$n_{21} = 1.254$
$\alpha_{11,palm} = 0.122$	$k_{22} = 0.583\ \tau^{-1}$
$\alpha_{11,\mathrm{T2D}} = 0.095$	$k_{23} = 1.64 \cdot 10^{-11}\ \tau^{-1}$
$k_{12} = 3.87 \cdot 10^{-15}\ \tau^{-1}$	$k_{26} = 6.25 \cdot 10^{-13}\ \tau^{-1}$
$k_{13} = 2.85 \cdot 10^{-10}\ \tau^{-1}$	$k_{27} = 7.89 \cdot 10^{-7}\ \tau^{-1}$
$k_{14,1}^{'} = 4.05 \cdot 10^{-14}\ x_{RNA}\ \ \tau^{-1}$	$k_{28} = 4.91 \cdot 10^{-5}\ \tau^{-1}$
$w_{14,1_0} = 1.96 \cdot 10^{-7}$	$k_{29,1} = 6.12 \cdot 10^{-14}\ \tau^{-1}$
$w_{14,1_5} = 2.23 \cdot 10^{-4}$	$k_{29,2} = 2.77 \cdot 10^{-4}\ \tau^{-1}$
$w_{14,1_8} = 6.84 \cdot 10^{-5}$	$k_{30} = 4.37 \cdot 10^{-14}\ \tau^{-1}$
$r_{14,1_{58}} = 9.07 \cdot 10^{-8}$	$x_{pr} / x_{RNA} = 35958$
$K_{14,1_5} = 4.99 \cdot 10^{-8}\ x_{pr}$	

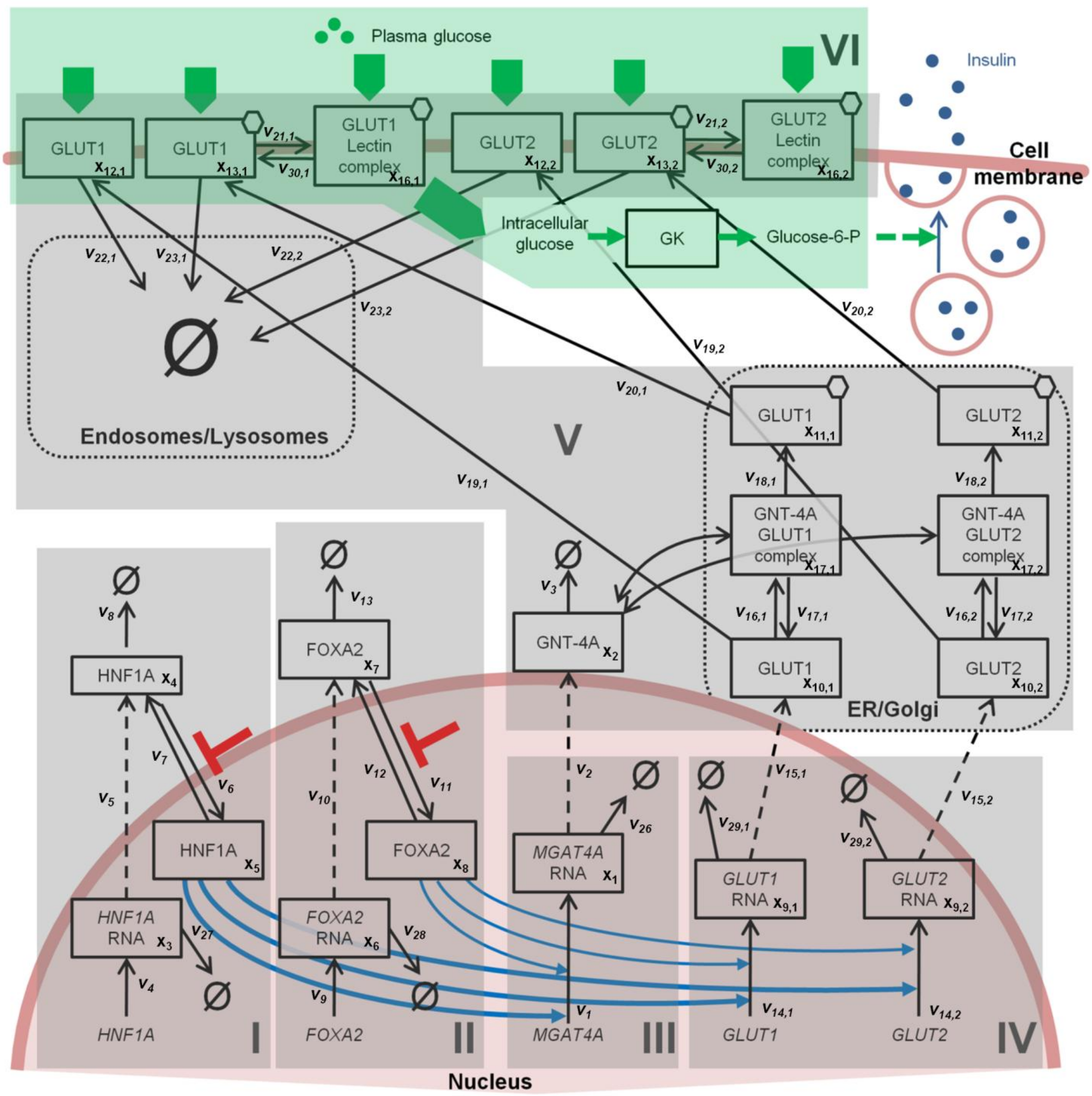

Figure S1. Same as Figure 3, with state variable and kinetic expression nomenclature indicated.

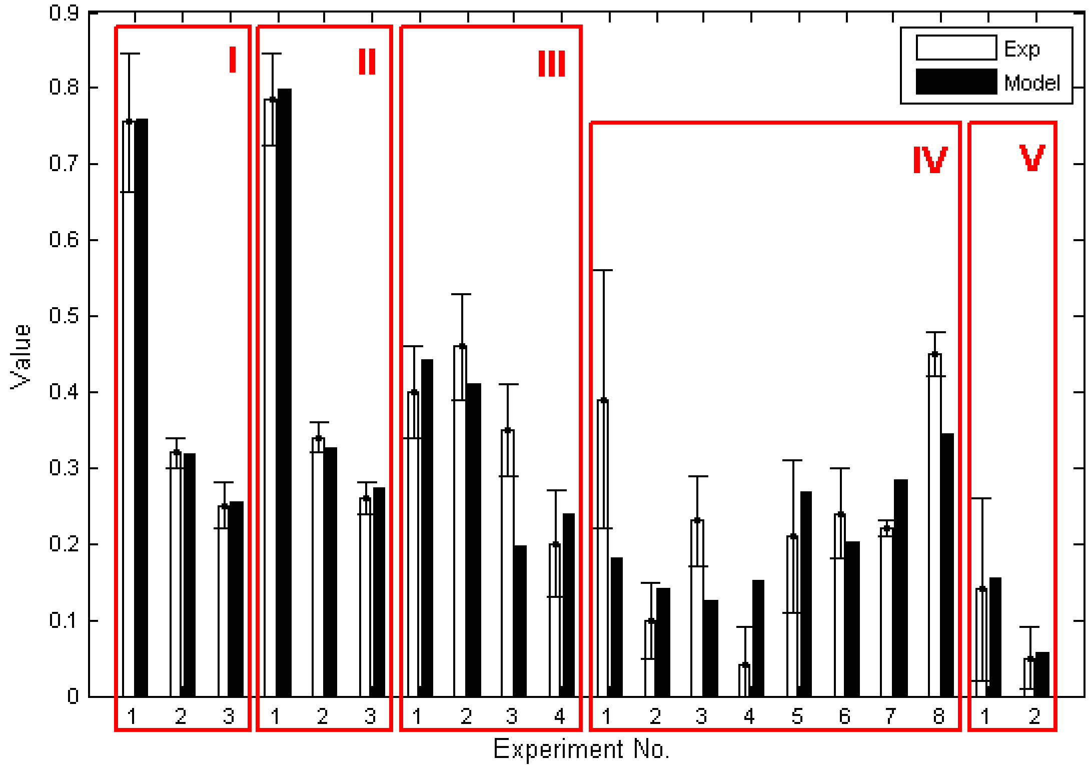

Figure S2. Comparison of experimental data described in Table S3 and model simulation results. Error bars represent the standard deviation of the data. Roman numbers refer to the subsystems highlighted in Figure 3 in the Main text.

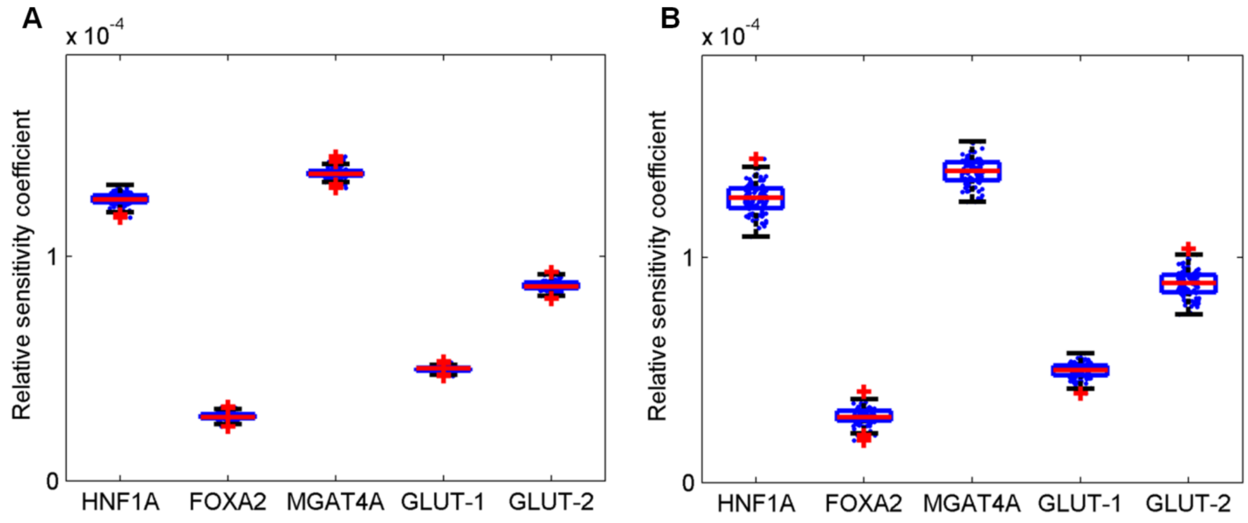

Figure S3. Robustness of local sensitivity analysis. Steady-state sensitivity of GK rate in T2D cells with respect to elevation in the RNA abundance of the genes indicated, at extra-cellular glucose concentration of 16.8 mM. Blue dots represent the relative sensitivity coefficients obtained from 100 random changes of one (A) or 10 (B) parameter values. Box plots summarize the statistical analysis of the results.

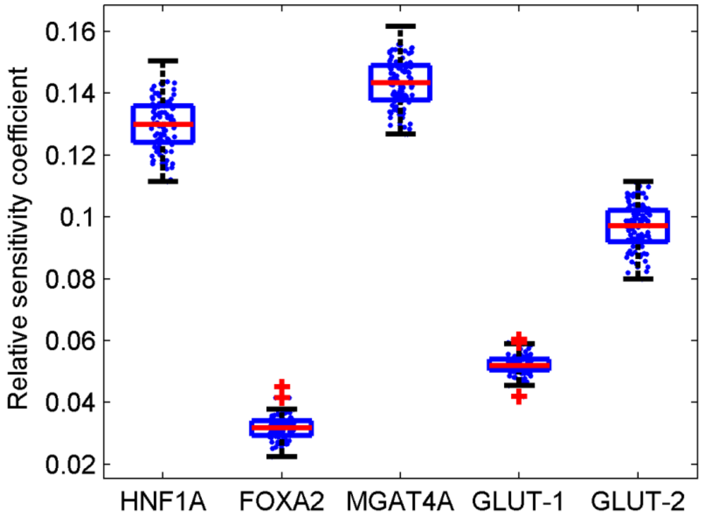

Figure S4. Effect of large parameter variations on the sensitivity analysis. Steady-state sensitivity of GK rate in T2D cells with respect to 25% elevation in the RNA abundance of the genes indicated, at extra-cellular glucose concentration of 16.8 mM. Blue dots represent the relative sensitivity coefficients obtained from 100 random changes of 10 parameter values. Box plots summarize the statistical analysis of the results.
