# Supplementary material for: Computational Modeling of Glucose Transport in Pancreatic β-Cells Identifies Metabolic Thresholds and Therapeutic Targets in Diabetes
Source: PLoS One. 2012 Dec 27;7(12):e53130. doi: 10.1371/journal.pone.0053130 (PMC3531366; doi:10.1371/journal.pone.0053130)
Supplement: Text S3 — MATLAB programs of the full model. (PDF) [file pone.0053130.s003.pdf]

Supplemental Information, Text S3

Luni C, Marth JD, Doyle III FJ. Computational Modeling of Glucose Transport in Pancreatic β-cells Identifies Metabolic Thresholds and Therapeutic Targets in Diabetes. *PLOS One*.

```
clear all; close all; clc
format long g
%% Module I-V
par0 = Parameters;
[mGLUT1_norm,mGLUT2_norm,mGLUT1_T2D,mGLUT2_T2D] = Model_basal_Nominal(par0);
epsilon1_T2D = mGLUT1_T2D/mGLUT1_norm;
epsilon2_T2D = mGLUT2_T2D/mGLUT2_norm;
%% Module VI
Vmax1h = par0(60); % uM/min
KD1 = par0(61); % mM
Vmax2h = par0(62);
KD2 = par0(63);
Vmax_GK = par0(64);
KM = par0(65);
nH = par0(66);
Vmax1h = Vmax1h*1e3; % nM/min
Vmax2h = Vmax2h*1e3;
Vmax_GK = Vmax_GK*1e3;
Ncell = 1e5;
Rcell = 10e-5; % dm
Vcell = 4/3*pi*Rcell^3; % [l/cell] OK 4.2 pl/cell
Vmax1h = Vmax1h*Vcell*Ncell; % nmol/min/10^5 cell
Vmax2h = Vmax2h*Vcell*Ncell; % nmol/min/10^5 cell
Vmax_GK = Vmax_GK*Vcell*Ncell; % nmol/min/10^5 cell
glucoseOFF = 2.8; % mM
glucoseON = 16.8; % mM
% Normal
epsilon1 = 1;
epsilon2 = 1;
Vmax1 = Vmax1h*epsilon1; % nmol/min/10^5 cell
Vmax2 = Vmax2h*epsilon2; % nmol/min/10^5 cell
[t,x] = Simulation(Vmax1,Vmax2,Vmax_GK,KD1,KD2,KM,nH,glucoseOFF,glucoseON);
t_norm = t;
g_e = (glucoseON-glucoseOFF)*(tanh(t-10)+1)/2+glucoseOFF; % mM
g_i_norm = x(:,1); % mM
g_P_norm = x(:,2); % mM
v1_norm = Vmax1*g_e./(KD1+g_e); % nmol/min/10^5 cell
v_1_norm = Vmax1*g_i_norm./(KD1+g_i_norm); % nmol/min/10^5 cell
uptake1_norm = v1_norm-v_1_norm; % nmol/min/10^5 cell
v2_norm = Vmax2*g_e./(KD2+g_e); % nmol/min/10^5 cell
v_2_norm = Vmax2*g_i_norm./(KD2+g_i_norm); % nmol/min/10^5 cell
uptake2_norm = v2_norm-v_2_norm; % nmol/min/10^5 cell
v_GK_norm = Vmax_GK*g_i_norm.^nH./(KM^nH+g_i_norm.^nH); % nmol/min/10^5 cell
% T2D
epsilon1 = epsilon1_T2D;
epsilon2 = epsilon2_T2D;
Vmax1 = Vmax1h*epsilon1; % nmol/min/10^5 cell
Vmax2 = Vmax2h*epsilon2; % nmol/min/10^5 cell
[t,x] = Simulation(Vmax1,Vmax2,Vmax_GK,KD1,KD2,KM,nH,glucoseOFF,glucoseON);
t_T2D = t;
g_e = (glucoseON-glucoseOFF)*(tanh(t-10)+1)/2+glucoseOFF; % mM
g_i_T2D = x(:,1); % mM
g_P_T2D = x(:,2); % mM
v1_T2D = Vmax1*g_e./(KD1+g_e); % nmol/min/10^5 cell
v_1_T2D = Vmax1*g_i_T2D./(KD1+g_i_T2D); % nmol/min/10^5 cell
uptake1_T2D = v1_T2D-v_1_T2D; % nmol/min/10^5 cell
v2_T2D = Vmax2*g_e./(KD2+g_e); % nmol/min/10^5 cell
```

```
v_2_T2D = Vmax2*g_i_T2D./(KD2+g_i_T2D); % nmol/min/10^5 cell
uptake2_T2D = v2_T2D-v_2_T2D; % nmol/min/10^5 cell
v_GK_T2D = Vmax_GK*g_i_T2D.^nH./(KM^nH+g_i_T2D.^nH); % nmol/min/10^5 cell
%% Figure
figure(1);
subplot 321
plot(t_T2D,g_e,'k','LineWidth',2)
ylabel('Plasma glucose')
subplot 322
plot(t_norm,g_i_norm,'k','LineWidth',2)
hold on
plot(t_T2D,g_i_T2D,'Color',[0.7 0.7 0.7],'LineWidth',2)
ylabel('Intracellular glucose')
subplot 323
plot(t_norm,uptake1_norm,'k','LineWidth',2)
hold on
plot(t_T2D,uptake1_T2D,'Color',[0.7 0.7 0.7],'LineWidth',2)
ylim([0 0.14])
ylabel('GLUT1 uptake')
subplot 324
plot(t_norm,uptake2_norm,'k','LineWidth',2)
hold on
plot(t_T2D,uptake2_T2D,'Color',[0.7 0.7 0.7],'LineWidth',2)
ylim([0 0.5])
ylabel('GLUT2 uptake')
subplot 325
plot(t_norm,v_GK_norm,'k','LineWidth',2)
hold on
plot(t_T2D,v_GK_T2D,'Color',[0.7 0.7 0.7],'LineWidth',2)
ylim([0 0.12])
xlabel('Time, (min)')
ylabel('GK rate')
subplot 326
plot(t_norm,g_P_norm,'k','LineWidth',2)
hold on
plot(t_T2D,g_P_T2D,'Color',[0.7 0.7 0.7],'LineWidth',2)
ylim([0 5])
xlabel('Time, (min)')
ylabel('glucose-P')

function par0 = Parameters
k4 = 4.16e-12;
k5 = 4.69e-12;
k6 = 0.016;
k7 = 5.14e-3;
k8 = 6.85e-8;
k27 = 7.89e-7;
alpha6_palm = 0.148;
alpha6_T2D = 0.109;
k9 = 3.45e-19;
k10 = 7.08e-16;
k11 = 1.53e-14;
k12 = 3.87e-15;
k13 = 2.85e-10;
k28 = 4.91e-5;
alpha11_palm = 0.122;
alpha11_T2D = 0.095;
k1_1 = 6.17e-6;
k26 = 6.25e-13;
w1_0 = 1.79e-11;
w1_5 = 1.79e-20;
w1_8 = 0.033;
r1_58 = 2.55e-9;
```

```
K1_5 = 1.40e-9;
K1_8 = 3.04e-11;
k1_2 = 1.11e-11;
k14_1p = 4.05e-14;
k29p = 6.12e-14;
w14_0p = 1.96e-7;
w14_5p = 2.23e-4;
w14_8p = 6.84e-5;
r14_58p = 9.07e-8;
K14_5p = 4.99e-8;
K14_8p = 1.91e-10;
k14_2p = 4.02e-12;
k14_1s = 2.27e-8;
k29s = 2.77e-4;
w14_0s = 6.57e-11;
w14_5s = 6.10e-14;
w14_8s = 1.27e-11;
r14_58s = 9.35e-19;
K14_5s = 1.51e-8;
K14_8s = 3.88e-20;
k14_2s = 1.09e-5;
k2 = 2.58e-15;
k3 = 1.23e-17;
k15p = 2.54e-13;
k15s = 3.11e-1;
k16 = 8.87e-16;
k17 = 5.72e-2;
k18 = 6.36e-10;
k19 = 3.30e-10;
k20 = 2.46e-5;
k21_1 = 6.98e-16;
k21_2 = 1.6e-18;
n21 = 1.254;
k22 = 0.583;
k23 = 1.64e-11;
k30 = 4.37e-14;
x_prot_xRNA = 35958;
Vmax1h = 1059.54; % uM/min
KD1 = 3; % mM
Vmax2h = 3910.51;
KD2 = 17;
Vmax_GK = 420.17;
KM = 8;
nH = 1.7;
par0 = [k4 k5 k6 k7 k8 k27 alpha6_palm alpha6_T2D ...
    k9 k10 k11 k12 k13 k28 alpha11_palm alpha11_T2D ...
    k1_1 k26 w1_0 w1_5 w1_8 r1_58 K1_5 K1_8 k1_2 ...
    k14_1p k29p w14_0p w14_5p w14_8p r14_58p K14_5p K14_8p k14_2p ...
    k14_1s k29s w14_0s w14_5s w14_8s r14_58s K14_5s K14_8s k14_2s ...
    k2 k3 k15p k15s k16 k17 k18 k19 k20 k21_1 k21_2 n21 k22 k23 k30 x_prot_xRNA, ...
    Vmax1h KD1 Vmax2h KD2 Vmax_GK KM nH];

function [mGLUT1_norm,mGLUT2_norm,mGLUT1_T2D,mGLUT2_T2D] = Model_basal_Nominal(par0)
k4 = par0(1);
k5 = par0(2);
k6 = par0(3);
k7 = par0(4);
k8 = par0(5);
k27 = par0(6);
alpha6_T2D = par0(8);
k9 = par0(9);
k10 = par0(10);
k11 = par0(11);
```

```
k12 = par0(12);
k13 = par0(13);
k28 = par0(14);
alpha11_T2D = par0(16);
k1_1 = par0(17);
k26 = par0(18);
w1_0 = par0(19);
w1_5 = par0(20);
w1_8 = par0(21);
r1_58 = par0(22);
K1_5 = par0(23);
K1_8 = par0(24);
k1_2 = par0(25);
k14_1p = par0(26);
k29p = par0(27);
w14_0p = par0(28);
w14_5p = par0(29);
w14_8p = par0(30);
r14_58p = par0(31);
K14_5p = par0(32);
K14_8p = par0(33);
k14_2p = par0(34);
k14_1s = par0(35);
k29s = par0(36);
w14_0s = par0(37);
w14_5s = par0(38);
w14_8s = par0(39);
r14_58s = par0(40);
K14_5s = par0(41);
K14_8s = par0(42);
k14_2s = par0(43);
k2 = par0(44);
k3 = par0(45);
k15p = par0(46);
k15s = par0(47);
k16 = par0(48);
k17 = par0(49);
k18 = par0(50);
k19 = par0(51);
k20 = par0(52);
k21_1 = par0(53);
k21_2 = par0(54);
n21 = par0(55);
k22 = par0(56);
k23 = par0(57);
k30 = par0(58);
x_prot_xRNA = par0(59);
%% Adimensionalization factors
tau = 1;
x_RNA = 1;
x_prot = x_prot_xRNA*x_RNA;
%% Derived parameters
w1_58 = 1-w1_0-w1_5-w1_8;
if or(w1_58 < 0, w1_58 > 1)
    Error_w1_58
end
w14_58p = 1-w14_0p-w14_5p-w14_8p;
if or(w14_58p < 0, w14_58p > 1)
    Error_w14_58p
end
w14_58s = 1-w14_0s-w14_5s-w14_8s;
if or(w14_58s < 0, w14_58s > 1)
    Error_w14_58s
```

```
end
%% Dimensional parameters
% Subsystem I: HNF
k4d = k4/tau*x_RNA;
k5d = k5/tau*x_prot/x_RNA;
k6d = k6/tau;
k7d = k7/tau;
k8d = k8/tau;
k27d = k27/tau;
alpha6_T2Dd = alpha6_T2D;
% Subsystem II: Foxa2
k9d = k9/tau*x_RNA;
k10d = k10/tau*x_prot/x_RNA;
k11d = k11/tau;
k12d = k12/tau;
k13d = k13/tau;
k28d = k28/tau;
alpha11_T2Dd = alpha11_T2D;
% Subsystem III: Mgat4a
k1_1d = k1_1/tau*x_RNA;
k26d = k26/tau;
w1_0d = w1_0;
w1_5d = w1_5;
w1_8d = w1_8;
w1_58d = w1_58;
r1_58d = r1_58;
K1_5d = K1_5*x_prot;
K1_8d = K1_8*x_prot;
k1_2d = k1_2*x_prot;
% Subsystem IV-p: Glut1
k14_1pd = k14_1p/tau*x_RNA;
k29pd = k29p/tau;
w14_0pd = w14_0p;
w14_5pd = w14_5p;
w14_8pd = w14_8p;
w14_58pd = w14_58p;
r14_58pd = r14_58p;
K14_5pd = K14_5p*x_prot;
K14_8pd = K14_8p*x_prot;
k14_2pd = k14_2p*x_prot;
% Subsystem IV-s: Glut2
k14_1sd = k14_1s/tau*x_RNA;
k29sd = k29s/tau;
w14_0sd = w14_0s;
w14_5sd = w14_5s;
w14_8sd = w14_8s;
w14_58sd = w14_58s;
r14_58sd = r14_58s;
K14_5sd = K14_5s*x_prot;
K14_8sd = K14_8s*x_prot;
k14_2sd = k14_2s*x_prot;
% Subsystem V: proteins
k2d = k2/tau*x_prot/x_RNA;
k3d = k3/tau;
k15pd = k15p/tau*x_prot/x_RNA;
k15sd = k15s/tau*x_prot/x_RNA;
k16d = k16/tau;
k17d = k17/tau;
k18d = k18/tau;
k19d = k19/tau;
k20d = k20/tau;
k21_1d = k21_1/tau*x_prot;
k21_2d = k21_2*x_prot;
```

```
n21d = n21;
k22d = k22/tau;
k23d = k23/tau;
k30d = k30/tau;
%% Model output: steady-states
% Subsystem I: HNF
x5ss_norm = (k4d*k5d*k6d)/(k27d*k7d*k8d);
x5ss_T2D = (alpha6_T2Dd*k4d*k5d*k6d)/(k27d*k7d*k8d);
% Subsystem I: Foxa2
x8ss_norm = (k9d*k10d*k11d)/(k28d*k13d*k12d);
x8ss_T2D = (alpha11_T2Dd*k9d*k10d*k11d)/(k28d*k13d*k12d);
% Subsystem III: Mgat4a
den_norm = 1+x5ss_norm/K1_5d+x8ss_norm/K1_8d+r1_58d*x5ss_norm/K1_5d*x8ss_norm/K1_8d;
phi_norm =
(w1_0d+w1_5d*x5ss_norm/K1_5d+w1_8d*x8ss_norm/K1_8d+w1_58d*r1_58d*x5ss_norm/K1_5d*x8ss_nor
m/K1_8d)/den_norm;
x1ss_norm = k1_1d/k26d*phi_norm*x5ss_norm/(k1_2d+x5ss_norm);
den_T2D = 1+x5ss_T2D/K1_5d+x8ss_T2D/K1_8d+r1_58d*x5ss_T2D/K1_5d*x8ss_T2D/K1_8d;
phi_T2D =
(w1_0d+w1_5d*x5ss_T2D/K1_5d+w1_8d*x8ss_T2D/K1_8d+w1_58d*r1_58d*x5ss_T2D/K1_5d*x8ss_T2D/K1
_8d)/den_T2D;
x1ss_T2D = k1_1d/k26d*phi_T2D*x5ss_T2D/(k1_2d+x5ss_T2D);
% Subsystem IV-p: Glut1
den_norm =
1+x5ss_norm/K14_5pd+x8ss_norm/K14_8pd+r14_58pd*x5ss_norm/K14_5pd*x8ss_norm/K14_8pd;
phi_norm =
(w14_0pd+w14_5pd*x5ss_norm/K14_5pd+w14_8pd*x8ss_norm/K14_8pd+w14_58pd*r14_58pd*x5ss_norm/
K14_5pd*x8ss_norm/K14_8pd)/den_norm;
x9pss_norm = k14_1pd/k29pd*phi_norm*x5ss_norm/(k14_2pd+x5ss_norm);
den_T2D = 1+x5ss_T2D/K14_5pd+x8ss_T2D/K14_8pd+r14_58pd*x5ss_T2D/K14_5pd*x8ss_T2D/K14_8pd;
phi_T2D =
(w14_0pd+w14_5pd*x5ss_T2D/K14_5pd+w14_8pd*x8ss_T2D/K14_8pd+w14_58pd*r14_58pd*x5ss_T2D/K14
_5pd*x8ss_T2D/K14_8pd)/den_T2D;
x9pss_T2D = k14_1pd/k29pd*phi_T2D*x5ss_T2D/(k14_2pd+x5ss_T2D);
% Subsystem IV-s: Glut2
den_norm =
1+x5ss_norm/K14_5sd+x8ss_norm/K14_8sd+r14_58sd*x5ss_norm/K14_5sd*x8ss_norm/K14_8sd;
phi_norm =
(w14_0sd+w14_5sd*x5ss_norm/K14_5sd+w14_8sd*x8ss_norm/K14_8sd+w14_58sd*r14_58sd*x5ss_norm/
K14_5sd*x8ss_norm/K14_8sd)/den_norm;
x9sss_norm = k14_1sd/k29sd*phi_norm*x5ss_norm/(k14_2sd+x5ss_norm);
den_T2D = 1+x5ss_T2D/K14_5sd+x8ss_T2D/K14_8sd+r14_58sd*x5ss_T2D/K14_5sd*x8ss_T2D/K14_8sd;
phi_T2D =
(w14_0sd+w14_5sd*x5ss_T2D/K14_5sd+w14_8sd*x8ss_T2D/K14_8sd+w14_58sd*r14_58sd*x5ss_T2D/K14
_5sd*x8ss_T2D/K14_8sd)/den_T2D;
x9sss_T2D = k14_1sd/k29sd*phi_T2D*x5ss_T2D/(k14_2sd+x5ss_T2D);
% Subsystem V: proteins
x2ss_norm = -(k17d*k19d*k3d + k18d*k19d*k3d - k16d*k18d*k2d*x1ss_norm +
k15pd*k16d*k18d*x9pss_norm + k15sd*k16d*k18d*x9sss_norm - ...
    sqrt(4*k16d*k18d*(k17d + k18d)*k19d*k2d*k3d*x1ss_norm + (k17d*k19d*k3d +
k18d*(k19d*k3d + k16d*(-(k2d*x1ss_norm) + k15pd*x9pss_norm +
k15sd*x9sss_norm)))^2))/(2*k16d*k18d*k3d);
x10pss_norm = k15pd*(k17d+k18d)*x9pss_norm/(k17d*k19d+k18d*(k19d+k16d*x2ss_norm));
x12pss_norm = k19d*x10pss_norm/k22d;
x17pss_norm = (k16d*x2ss_norm*x10pss_norm)/(k17d+k18d);
x11pss_norm = (k18d*x17pss_norm)/k20d;
x13pss_norm = (k20d*x11pss_norm)/k23d;
x16pss_norm = (k21_1d*x13pss_norm^n21d/(k21_2d^n21d+x13pss_norm^n21d))/k30d;
x10sss_norm = k15sd*(k17d+k18d)*x9sss_norm/(k17d*k19d+k18d*(k19d+k16d*x2ss_norm));
x12sss_norm = k19d*x10sss_norm/k22d;
x17sss_norm = (k16d*x2ss_norm*x10sss_norm)/(k17d+k18d);
x11sss_norm = (k18d*x17sss_norm)/k20d;
x13sss_norm = (k20d*x11sss_norm)/k23d;
```

```
x16sss_norm = (k21_1d*x13sss_norm^n21d/(k21_2d^n21d+x13sss_norm^n21d))/k30d;
mGLUT1_norm = x12pss_norm+x13pss_norm+x16pss_norm;
mGLUT2_norm = x12sss_norm+x13sss_norm+x16sss_norm;
x2ss_T2D = -(k17d*k19d*k3d + k18d*k19d*k3d - k16d*k18d*k2d*x1ss_T2D +
k15pd*k16d*k18d*x9pss_T2D + k15sd*k16d*k18d*x9sss_T2D - ...
    sqrt(4*k16d*k18d*(k17d + k18d)*k19d*k2d*k3d*x1ss_T2D + (k17d*k19d*k3d +
k18d*(k19d*k3d + k16d*(-(k2d*x1ss_T2D) + k15pd*x9pss_T2D +
k15sd*x9sss_T2D)))^2))/(2*k16d*k18d*k3d);
x10pss_T2D = k15pd*(k17d+k18d)*x9pss_T2D/(k17d*k19d+k18d*(k19d+k16d*x2ss_T2D));
x12pss_T2D = k19d*x10pss_T2D/k22d;
x17pss_T2D = (k16d*x2ss_T2D*x10pss_T2D)/(k17d+k18d);
x11pss_T2D = (k18d*x17pss_T2D)/k20d;
x13pss_T2D = (k20d*x11pss_T2D)/k23d;
x16pss_T2D = (k21_1d*x13pss_T2D^n21d/(k21_2d^n21d+x13pss_T2D^n21d))/k30d;
x10sss_T2D = k15sd*(k17d+k18d)*x9sss_T2D/(k17d*k19d+k18d*(k19d+k16d*x2ss_T2D));
x12sss_T2D = k19d*x10sss_T2D/k22d;
x17sss_T2D = (k16d*x2ss_T2D*x10sss_T2D)/(k17d+k18d);
x11sss_T2D = (k18d*x17sss_T2D)/k20d;
x13sss_T2D = (k20d*x11sss_T2D)/k23d;
x16sss_T2D = (k21_1d*x13sss_T2D^n21d/(k21_2d^n21d+x13sss_T2D^n21d))/k30d;
mGLUT1_T2D = x12pss_T2D+x13pss_T2D+x16pss_T2D;
mGLUT2_T2D = x12sss_T2D+x13sss_T2D+x16sss_T2D;

function [t,x] = Simulation(Vmax1,Vmax2,Vmax_GK,KD1,KD2,KM,nH,glucoseOFF,glucoseON)
% Glucose low
x0 = [0 0]; % [g_i g_P]
tspan = [0 1e4];
[t,x] = ODE_moduleVI(Vmax1,Vmax2,Vmax_GK,KD1,KD2,KM,nH,tspan,x0,glucoseOFF,glucoseOFF);
% Glucose step
x0 = [x(end,1),0];
tspan = [0 60];
[t,x] = ODE_moduleVI(Vmax1,Vmax2,Vmax_GK,KD1,KD2,KM,nH,tspan,x0,glucoseOFF,glucoseON);

function [t,x] =
ODE_moduleVI(Vmax1,Vmax2,Vmax_GK,KD1,KD2,KM,nH,tspan,x0,glucoseOFF,glucoseON)
[t,x] = ode15s(@myfun,tspan,x0);
    function dx = myfun(t,x)
        g_i = x(1); % mM
        g_P = x(2); % mM
        g_e = (glucoseON-glucoseOFF)*(tanh(t-10)+1)/2+glucoseOFF; % mM
        v1 = Vmax1*g_e/(KD1+g_e); % nmol/min/10^5 cell
        v_1 = Vmax1*g_i/(KD1+g_i); % nmol/min/10^5 cell
        v2 = Vmax2*g_e/(KD2+g_e); % nmol/min/10^5 cell
        v_2 = Vmax2*g_i/(KD2+g_i); % nmol/min/10^5 cell
        v_GK = Vmax_GK*g_i^nH/(KM^nH+g_i^nH); % nmol/min/10^5 cell
        dg_i = (v1-v_1)+(v2-v_2)-v_GK; % nmol/min/10^5 cell
        dg_P = v_GK; % nmol/min/10^5 cell
        dx = [dg_i; dg_P];
    end
end
```
